# ‘Stranger Views’: Researching Marginality and (Non)Belonging Among Migrants Experiencing Homelessness in the UK

**DOI:** 10.1111/1468-4446.70033

**Published:** 2025-09-28

**Authors:** Simon Stewart, Marianela Barrios Aquino

**Affiliations:** ^1^ Centre for European and International Studies Research Faculty of Humanities and Social Sciences University of Portsmouth Portsmouth UK

**Keywords:** marginality, migrant homelessness, migration, New Area Studies, Simmel, stranger

## Abstract

With reference to Simmel's work, this article puts forward the notion of ‘stranger views’, which are expressive on the one hand, of the experiences of those who occupy a marginal position in society characterised by experiences of belonging and non‐belonging, and on the other, of our own position as researchers, probing spaces of non‐belonging and hearing stories that are then rearticulated for an academic audience. In doing so, it provides a reflective dialog between the findings of a research project on migrant homelessness in the UK and the methodological framework brought by New Area Studies. The article deploys the life story research method and focuses on views of the UK from the perspective of migrants from former European colonies who have been in the UK for several years but whose immigration status and lack of economic capital renders them vulnerable to destitution and homelessness. The article offers unique insights into the co‐existence of belonging and non‐belonging and the dissonance between these feelings. In providing a dialog between accounts deriving from life story interviews with migrants experiencing homelessness and a self‐critical reflection about the knowledge produced with such accounts, our article contributes to debates on the sociology of marginality with a three‐tiered discussion of migration, homelessness and methodological frameworks, which are rarely considered together.

## 1 Introduction

Consideration of the relationship between power and knowledge is crucial for our present reflection on marginality and migrant homelessness. Following Chimni (2009) we understand the double nature of knowledge, as it can serve forces both of ‘dominance and emancipation’ (p. 14), serving to legitimise subordination and work for the empowerment of marginal groups. In this article, we argue that self‐reflection is a precondition for critical engagement with the relationship between power and knowledge, and more specifically, between marginalised voices and the study of marginality.

As Wacquant (2008, 231) observes in his analyses of the French working‐class periphery and an African‐American neighbourhood, each instance of marginality needs to be considered in relation to its specific historical situation, bearing in mind, for example, the complex interplay between class, race and the nation state. Nevertheless, Wacquant proceeds to argue that there are forms of what he terms *advanced marginality* that have much in common. Key characteristics of this phenomenon include forms of social closure that effectively block dominated groups from accessing key societal networks, resources and the ability to compete for these resources. Whereas in his study, Wacquant focuses on peripheries and deprived areas, our article focuses on a project led by Stewart (Stewart et al. 2023; Stewart and Sanders 2023), which researches the lives of migrants experiencing homelessness whose lives are not fixed to specific neighbourhoods and who have a greater need to move around the city, disconnected as they are from secure accommodation and access to the job market.

In this article, we extend Simmel's (1971[1908]) discussion of the social type of *the stranger* to apply it to the cases of migrants experiencing homelessness in the UK and to ourselves as researchers of marginalities. With a focus on migrants from former European colonies, we demonstrate how their experiences of marginality, simultaneously belonging and non‐belonging enables ‘stranger views’, that is insights into the dominant society from the perspective of the dominated. These stranger views are expressive of the co‐existence of feelings of belonging and non‐belonging. Despite the suffering experienced by many of the research participants before coming to the UK and during their time here, where they have experienced homelessness, the perspectives that they offer about life in the UK provide unique insights from the point of view of those who have often been in the country for many years. Their stories convey a sense of being part of life in the UK, and yet as a consequence of their immigration status and declining material conditions of existence, provide unsettling reminders of their non‐belonging. We explore the dissonance between their senses of belonging and non‐belonging.

Simultaneously, we conceptualise our views as ‘stranger views’, highlighting the power dynamics between us and our research participants. Thus, at the core of the article is a reflective dialogue between the knowledge produced by Stewart's project and the methodological framework brought by New Area Studies, which calls for more ethical and power critical research on issues related to marginality.

In providing a dialogue between accounts deriving from life story interviews with marginalised individuals from former European colonies and a self‐critical reflection about the knowledge produced with such accounts, our article contributes to debates with a three‐tiered discussion of migration, homelessness and methodological frameworks, which are rarely considered together. The article also innovates by drawing on approaches in New Area Studies that explore the potential of critical engagement with our own research on marginality in appraising systems of domination from the perspective of the dominated. New Area Studies, in this article, constitutes a way of doing research and thinking about research topics and is particularly useful to highlight the challenges researchers face when studying issues of marginality and the power dynamics that emerge from their research contexts and topics. Writing ourselves as researchers into the stories that we want to examine tests the conceptual frameworks that our disciplines rest upon and stretches the possibilities of critical thinking. In this case, we stretch Simmel's concept of the stranger, to simultaneously provide a sociological analysis of migrants' experiences of homelessness in the UK, and a reflexive account of research on homelessness.

## 2 Stranger Views and (Non)Belonging

Marginality is associated with a dominated social position occupied by individuals and groups where they are denied access to resources or the means of competition for resources. According to Wacquant (2008), the inter‐related characteristics of advanced marginality include insecure wage labour, disconnection from macroeconomic trends (even during times of economic prosperity), territorial stigmatisation, the dissolution of a sense of ‘place’, the loss of hinterland, and social fragmentation. Marginality is *advanced* because it is not an aspect of the past that is behind us, a residual issue that is in the process of being resolved. It is instead something that is currently taking shape, which looms on the horizon, and has consequences that lie ahead of us in the current moment. However, marginality is more than a site of domination, where inequalities are perpetuated, and where the dominant are in the strongest position to continue their plundering. For example, hooks argues that marginality is also a site of resistance and radical possibility (1991, p. 23). This has particular implications for us as social scientists, because it directs us to think about research produced from the margins as well as research produced about the margins. A focus on marginality as resistance invites us to engage in more power‐critical research, meaning that we ought to consider about whom knowledge is being produced and by whom (Barrios Aquino 2024). Following Foucault (1982), we can understand resistance as a diagnosis of power and not external or opposed to it. Similarly to hooks, Foucault proposes the use of resistance ‘to bring to light power relations, locate their position, find out their points of application and the methods used’ (1982, pp. 209–211). The point here is that marginality as resistance affords us the possibility of a critical approach where counter‐hegemonic practices and discourses reveal the sites of power and oppression and, simultaneously, provide opportunities for contestation and cultural change (hooks 1989, p. 15).

hooks's analysis of alternative perceptions and practices deriving from the position of marginality brings to mind key literature from classical social theory on this topic. Simmel's (1971) famous essay on the social type of the stranger, who is part of the group but also outside of it, draws attention to someone whose marginal social position enables a sense of double consciousness. Simmel's analysis of the stranger is in line with more recent research in migration studies, which refutes any simplistic binary of either belonging or non‐belonging, being inside or outside. Simmel argues that the stranger is part of the group and yet is separate from it. The stranger is someone who ‘comes today and stays tomorrow’; someone who is close to the group and yet remote (Simmel 1971, 143). In this article, we further undermine this binary opposition and pursue a definition of non‐belonging as much more than the absence of belonging. According to Korteweg and Yurdakul (2024, 294) non‐belonging ‘has its own logics and creates particular spaces’.

The tension between distance and closeness is a feature of all human interaction but in the social type of the stranger it takes a specific form. The stranger's interactions with others are characterised by the synthesis of belonging and non‐belonging and this position provides an alternative to the hegemonic views. The stranger's views also cast a light on the active production of non‐belonging through policies and law (Korteweg and Yurdakul 2024) which produce legal categories that in the case of migrants ‘are not merely devices for inclusion but also exclusion’ (Chimni 2009, 12). The legal categories ascribed to the stranger (in this case referring to those with ‘inferior’ immigration statuses) have meant that they are more likely than others to get the blame when things go wrong and are thus vulnerable to stereotyping and forms of harmful representation. Moreover, these legal categories result in a colonial hierarchy of human bodies that continues to shape the politics of belonging today (Wynter 2003; Yuval‐Davis 2006).

Inspired by Simmel's (1971) essay and by Korteweg and Yurdakul’s (2024) conceptualisation of colonial spaces of non‐belonging, we consider the position of migrants experiencing homelessness in the UK as examples of advanced marginality and engage with it as an opportunity to scrutinise knowledge production about marginalised communities. The social problem of migrants experiencing homelessness, in the context of an increasingly hostile immigration environment in the UK, is not a residual issue from a previous era that is in the process of being resolved. In line with Wacquant’s (2008, 232) argument, it is very much part of the present moment and looms on the horizon. Under successive governments, migrants with No Recourse to Public Funds (NRPF) remain particularly vulnerable to destitution, with no access to social benefits, the job market or housing. Without secure housing, they are less likely to have access to a hinterland, a community or group on which they can depend during difficult times. Moreover, seeking out alternative means of survival, such as work in the informal economy or other forms of precarious work, entails a drift towards illegality and further disconnection from mainstream society (Stewart and Sanders 2023). As Wacquant (2008, 252) points out, the ‘free market’, which is one of the causes of advanced marginality, ‘can hardly be counted on to provide *remedies* for it’.

Drawing on Simmel, we argue that the position of simultaneous belonging and non‐belonging allows *stranger views* into life in the UK from the perspective of dominated groups. Our focus is on those experiencing a combination of material deprivation as homeless and carrying an ‘inferior’ position through being racialised as migrants and restricted in their access to rights taken for granted by UK citizens (Wynter 2003). Our article deploys the life story research method and focuses on views of the UK from the perspective of migrants from countries that were former European colonies, who have been in the UK for several years but whose immigration status and lack of economic capital renders them vulnerable to destitution. In doing so, the article contributes to the under‐researched topic of migrant homelessness. Each migrant experiencing homelessness occupies a unique space of non‐belonging combined with a sense of belonging. Some have lived in the UK for many decades while others have only recently arrived. What they have in common is the articulation of a stranger view of the UK: a perspective that on the one hand is expressive of familiarity and belonging, and moments of being part of the group, and on the other hand, one that reinforces their relegation to a space of non‐belonging that is most apparent when confronted with the reality of a weak structural position (e.g., lacking material resources and unable to access work or benefits) combined with the threat of deportation. This is a threat that looms even while it seems to be suspended. Stranger views offer insights from the inside and outside, casting a light on the dislocation that occurs when non‐belonging generates new modes of existence, where individuals are denied access to resources and/or the right to work and cling to the possibility of recovering rights that have been suspended (Korteweg and Yurdakul 2024; Stewart and Sanders 2024; Scuzzarello and Moroşanu 2023). But before we explore this further, we will turn to some reflections on marginality that underpin our approach to this topic.

## 3 Marginality, Power and Knowledge Production

*What* we research is deeply intertwined with *how* we do research. Marginality as a conceptual guide is a site of radical possibility to expand both our research *focus* as well as our research *practice*. Moreover, New Area Studies opens up methodological approaches for research practice that enable us to avoid reproducing the hierarchies we are trying to undermine (Barrios Aquino 2024).

Thus, in thinking through our epistemological approach, we are informed by debates in New Area Studies that interpret culture, politics and social relations in ways that are multi‐layered, non‐linear, made of complex temporalities and transcending borders, and where narratives and storytelling are key tools to access the rich variety of lifeworlds (Baumann and et al. 2020; Fleschenberg and Baumann 2020; Hutchings 2020; Hodgett and Ruys Smith 2021). Our reflection thus focuses on two aspects of marginality:

1. marginality in relation to spaces of (non)belonging as a guiding research concept, and
2. marginality in our knowledge production

Academic research on marginalities runs the risk of exotifying, othering and constructing cultures from the point of view of a western scientific perspective. This western scientific perspective refers here to a particular way of constructing a knowing subject ‘untouched by the geo‐political configuration of the world in which people are racially ranked and regions are racially configured’ (Mignolo 2009, 160). This involves the researcher who is arguably outside and looking in, adopting a stranger view with a very different inflection, and with the advantages of perspective, in our case afforded by the concepts, methods and resources available in Anglo‐American/European academia.

Relatedly, our self‐critical reflection is informed by feminist traditions and embraces our positionality, emotions and ethics as part of the process rather than seeking to bracket them out to gain ‘objectivity’ (Nadar 2014, 26). This results in an attempt to break the fiction of objectivity and neutrality and involve ourselves and our positionality in our research practice, in this case, even as a form of reflection after the research project has concluded. This situated knowledge follows a long‐standing tradition in feminist approaches to research (Rose 1997; Haraway 1988; Motta and Gonzalez 2023) and aims to open up spaces for the articulation of what Chimni (2009) termed the dual nature of knowledge, namely that it can be deployed for the purposes of emancipation and domination. With this in mind, we note the following:

1. Stranger views, as we research them, are able to offer insights into systems of domination from the perspective of those most impacted, from those who are simultaneously positioned as connected to and separated from the group. But also,
2. Researching stranger views has a double aspect because stranger views are also the views of researchers occupying positions outside of the marginalities they study. Therefore, as researchers, it is necessary to consider our own position vis‐à‐vis hegemonic systems of knowledge production.

As scholars in institutions in the Global North we need to ask ourselves questions that will lead to a justification of our research interests and the impact of our research practices. These are difficult questions to ask, as they imply a harsh reckoning with our place in the production of larger marginalities at a global scale (Odysseos 2017). With this in mind, we can assert the following:

1. The views of migrants experiencing homelessness provide an alternative to hegemonic views of life in the UK, constituting a powerful tool to understand the perpetuation of colonial hierarchies and the structural causes of homelessness and destitution; and
2. Our own knowledge production derivative of those views, as privileged researchers in the Global North who have never experienced insecure housing or been at risk of homelessness, risks reproducing such marginalities.

Because we are aware of the danger of reproducing marginalities, it is our responsibility to engage in an honest reflection about positionality and be fully aware of the spaces we occupy if we want to contribute to challenging dominant power relations and explore marginality as a site of possibility (Sadan 2020).

Simmel's notion of the stranger, as deployed in this article, is powerful in that it addresses two distinct positions in the context of research on marginality: first, that of those who occupy marginal spaces and are victims of their marginalisation (here, migrants experiencing homelessness); second, that of those who have access to that marginalisation from a position of power (us, as researchers). There is a tension between these two positions, where the stories told from the margins by those who are not on the margins are both valuable to identify social problems and simultaneously risk fixing the research participants' position in the margins. This begs the question: who is the stranger? This article therefore invites researchers to exercise reflexivity in considering the stranger views of the people and phenomena in the margins *and* those observing them and producing knowledge about their stories.

Insights can be taken from approaches in New Area Studies that call upon researchers to emphasise an understanding of the various ways in which our worldview is reproduced in our research objects. For example, as Barrios Aquino (2024) notes, hierarchies can be perpetuated through the research process when not attended to reflexively. There are many challenges in research of this kind. Given the power differential between the researcher and the researched, there is a danger that a project such as ours is merely extractive in treating migrants' accounts as data that can be used for our own career enhancement thus reproducing a colonial or capitalist logic. Further, there is a danger of retraumatising research participants by encouraging them to discuss potentially traumatic past experiences.

Through reflections that we encounter in discussions on New Area Studies, we have come to take more seriously the power dynamics at play in our relationship to the migrants experiencing homelessness that participated in Stewart's research project. Without these reflections, researchers risk becoming invisible and assuming a position of ‘scientific objectivity’, theorising homelessness as a characteristic of research participants and theorising their lives and experiences through dominant knowledge systems (academic research and language) (Omodan 2024). This is a colonial model of understanding the world that, if left unchallenged, perpetuates inequalities and places us (academics based in the West) as the holders of the truths.

## 4 Methodology

Stewart and colleagues chose the life story research method because it provided the benefit of opening up opportunities for insight into how individuals have responded to situations where their actions and choices are constrained by ‘the unequal structure of possibilities’ (Souza and Lemos 2023, 5), such as when they proceed with a lack of material resources and an ‘inferior’ immigration status. Moreover, the authors chose to engage with a reflection on knowledge production after the research project had finished, using New Area Studies as an epistemological framework. In this article, therefore, we engage both with ethics of qualitative research on marginalities and with a post‐research design reflection about our work as researchers thinking through marginality.

### 4.1 Research Design

Stewart's research was conducted between 2020 and 2022 and was funded by UK Research and Innovation and the Economic and Social Research Council.^1^ The research questions that structured the project were designed to explore migrants' experiences of homelessness before and during the COVID‐19 pandemic. The research team worked with nine homelessness organisations in the sector across three UK cities in the South of England, including London. Partnering with these organisations was central to the development of fieldwork, not only because of their expertise but also because they were key to putting the research team in contact with research participants. In formulating the project, Stewart worked with a homelessness organisation and through them, was able to contact staff and their clients at this and other organisations in the sector. The research was conducted in two phases: First, 37 semi‐structured interviews were carried out with staff and volunteers to get a better understanding of the context, that is, the situation of migrant homelessness. The fieldwork took place during the COVID‐19 pandemic when the country went in and out of lockdown, and interviews were conducted in‐person, online and over the phone. Paradoxically, the pandemic enabled access to those who were ordinarily finding ways to survive without statutory support because the UK Government's Everyone In initiative ensured that people were given homelessness assistance regardless of their immigration status (Stewart et al. 2023). In the second phase of the research, the research team conducted life story interviews with 43 migrants experiencing homelessness. All were non‐UK nationals who were born outside of the UK. These interviews were generally conducted over three sittings and were led by Stewart (the Principal Investigator) and members of the project team including the Co‐Investigator, the postdoctoral research associate and a colleague from the homelessness organisation who then worked on the project as an independent researcher.

The project team analysed the data thematically, pursuing a collaborative form of analysis which involved discussing the data and generating themes (Richards and Hemphill 2018). A key theme generated from the analysis of the data was the co‐existence of belonging and non‐belonging and the dissonance between these feelings among migrants experiencing homelessness in the UK. In this article, we have chosen to focus on vignettes based on the life stories of four of the research participants, all of whom hail from former European colonies (Albania, Algeria, Singapore and Sudan). These stories have been chosen because they offer striking illustrations of this theme of belonging and non‐belonging generated through the collaborative analysis. Our extended exploration of this selection of stories provides the rich detail necessary to understand the ambivalences in their accounts as well as the dis/continuities in the participants' experiences of migration and homelessness.

### 4.2 Life Stories

Life story interviews were chosen because they provide opportunities for diagnoses of systems of domination from the perspective of those most impacted. Further, the research project aimed to explore perspectives on the impact of hostile environment policies ‘from below’, that is from those most impacted by these policies. Given the sensitive nature of the topic of experiences of extreme destitution and marginality, a more humane and intimate approach was necessary in order to have access to this aspect of human experience. Life stories present the opportunity for an intimacy appropriate to this context. According to Moreno Figueroa (2008, 76) ‘writing sociological research is the process of intimate listening’. Life stories constitute a way of honouring that intimacy. The first part of the interview used prompts about life in the present; the second focused on journeys to the UK; the third section focused on social origins.

Insights from life story research in black feminism also demonstrate the role that telling stories can play in community building and in developing the courage to transform social relations (Nadar 2014). In contrast to abstract theory building, Nadar (2014) argues that life stories provide opportunities to put a human face on the research process. Further, in privileging subjectivity, they are not so concerned with replicating with total accuracy exactly what happened as much as how events were experienced by people.

When dealing with a topic such as this, it is important to acknowledge that there are not one but multiple forms of marginalisation that intersect and compound each other. For example, marginalisation on the basis of immigration status, ‘race’, class, gender, sexuality, disability, geographic location, and (lack of) possession of various forms of capital, all contribute to shaping experiences of marginalisation that cannot be summarised or flattened under the label of ‘homeless migrant’. Therefore, for the purpose of this article, we focus on a broad understanding of marginality, recognising that marginalisation can be reinforced through the research process. That is, by conceptualising people as ‘homeless migrants’ we run the risk of naturalising structural inequalities. How can this be avoided (if at all)? The first step is to focus on our own positionalities as researchers, and our own relationships to power and resistance, even though this double exercise of an epistemological nature may complicate our understanding of our own research ‘objects’.

Stewart is Professor of Sociology at University of Portsmouth and is a UK national. During the empirical stage of the project, the social distance between researcher and the research participants was often made apparent in the course of interactions. For example, one participant assumed that Stewart's academic credentials meant that he was likely to have connections with the Home Office which he could use to resolve their immigration paperwork for them or for members of their families. Stewart had no such connections or influence and so expectations needed to be carefully managed.

Stewart is joined here by Barrios Aquino in a reflection on research about migrants, marginality and knowledge production. Barrios Aquino is a migrant herself, who has lived through a variety of statuses in the past (undocumented, student resident, temporary residency, permanent residency, citizenship, revocation of citizenship, restoration of citizenship). The contrast between their lived experiences in combination with the synergies in their research interests has provided the opportunity to critically reflect on the conditions of their roles in knowledge production.

### 4.3 Ethics

This research was conducted in accordance with the ethical principles outlined in the ESRC Framework for Research Ethics and by the University of Portsmouth Ethics Committee, which has adopted the UK Research Integrity Office Code of Practice and the Universities UK Concordat to Support Research Integrity. As the fieldwork involved human participants from vulnerable populations, the project team sought guidance on conducting the interviews and producing the outputs from a professor who is also chair and director of a refugee charity and has extensive experience of working with individuals who have experienced trauma. Accordingly, Stewart designed the fieldwork to ensure that ethical safeguards and confidentiality procedures were strictly followed. The research participants consented to share the data provided in their life stories. Nevertheless, in order to protect their identities, we have ensured that all names included in this article (and in other related research outputs) are pseudonyms. Moreover, we removed all identifying information relating to organisations, specific locations, formal and informal networks, and job titles. The application was approved by the ethics committee at University of Portsmouth and by the main homelessness organisation with whom we collaborated. Each research participant received an hourly living wage (in vouchers) as remuneration for their time.

## 5 Relevant Intersecting Themes: Life in the UK

In this section, we discuss two themes that, cumulatively, bring together some critical reflections on issues of marginality. These themes are ‘colonial visions of England’ and ‘experiences of the hostile environment’. To do so, we present accounts from research participants that highlight the marginal position of migrants experiencing homelessness, which in turn enables what we have termed stranger views: These are insights into life in the UK from a position in the margins characterised by a combination of belonging and non‐belonging.

### 5.1 Colonial Visions of England

How do we make sense of the accounts of life in the UK provided by our respondents when they valorise a country in which they have been treated so badly by the Home Office and where they have experienced homelessness? How do such attitudes coexist with more recent life‐changing experiences in dealing with the Home Office in the UK while seeking to resolve their immigration status? We note that their conception of the UK as a ‘fair’ and ‘civilised’ country jars against accounts of their experiences and the ways in which they had been treated by the Home Office and has colonial undertones. The coexistence of these narratives is expressive of a social position that is at once part of the group and outside of it.

Angelina's case highlights the double consciousness that comes from occupying the spaces of belonging and non‐belonging, a sense of what Du Bois (1903, 2) referred to as ‘always looking at one's self through the eyes of others, of measuring one's soul by the tape of a world that looks on in amused contempt and pity’. Angelina is a Sudanese woman of dual heritage in her fifties who has been in the UK for several years and gained refugee status after experiencing a period of homelessness while awaiting the resolution of her asylum claim. She observes that

[M]y skin is not white. I’m not brown. But in my country they do not consider me as a brown lady, as a white lady, but here, [the] British do not consider me as a white lady, they consider me as a brown lady.

Her experiences of being racialised speak to the colonial hierarchy of bodies she encountered in the UK. Her societal status is also context dependent. Angelina hails from an affluent middle‐class family and grew up in a large, spacious house with lots of land. In contrast, while in the UK, she experienced homelessness and lived in precarious accommodation, surviving on minimal funds while seeking asylum. Angelina's affluent background allowed her to travel to the UK as a tourist before migrating, which afforded her a double consciousness regarding her experience of the UK. This brings to light Bauman's (1998) distinction between the tourist as the affluent person who is able to move freely across borders and the vagabond who is either forced to move or unable to cross borders because of a lack of papers and money. In previous years, with money at her disposal, Angelina travelled on several occasions to European countries, including the UK, as a tourist. She would visit European cities for shopping trips. Her experience of the UK this time, as an asylum seeker, was quite different. Her view of the UK is also split between bitter recollections of the application process for asylum and her general view of the country as a ‘civilised’ place. She strikes a contrast between Sudan and the UK, highlighting the colonial discourses that place not only bodies, but also countries in a hierarchy of civilisations: ‘We are an under‐developed country and we came to the civilised country. You can feel the gap’.

Joshua's account of life in the UK also combines a sense of belonging and non‐belonging. He came to the UK from Singapore more than 3 decades ago, on a student visa. In common with other research participants, he often refers to an imperial understanding of England, placing it at the top of the hierarchy of civilisation:

I mean from Singapore we are very English or British orientated, probably because … everybody speaks English. So, from a young age I always wanted England. I used to admire English history, culture, the language, so I already wanted to go to England and do my higher studies—that’s the only reason why I didn’t go to Singapore University.

I think this country is one of the most civilised, cultured countries in the true sense … But a lot of foreigners, refugees, illegal immigrants enter this country … why do they come to a country like England of all the countries in Europe? Because this is a civilised country. No country can be perfect, I know that, but this is a civilised country. I also agree that the system here, the government here, has to be tough on immigration. You don’t come into my house and mess up my house. You come decently and live decently—I strongly believe in that.

I don’t think any other country can be easily comparable. But I’m talking to you, not because I’m living in this country, I want to get benefits—no. That’s the reason why [a] small country dominated one third to two thirds of the world. What’s good about it? That’s the reason I wanted to come here, see the universities, get a degree from here, [and] see … Europe. I wanted to educate myself, so I came here, and I was not wrong. This country is not perfect but it’s a wonderful country, wonderful people I would say.

Joshua is now in his seventies. When he was a child, Singapore was a British colony. The views and dreams that he speaks of are indicative of colonial legacies. He recalls singing ‘London Bridge is falling down’ at the age of six or seven, and was inspired by images of Oxford Street and Regent Street in school textbooks. As a young adult, after the country's independence, he listened to BBC radio services and was influenced by British music and sport. He liked the Beatles, The Animals and Cliff Richard and, with his peers, he regularly gathered round television sets to watch English football matches.

In common with Joshua, Pipo, a British‐Algerian man in his mid‐forties, holds a great respect for colonial institutions in Britain. He refers to the UK as a place where there is equality before the law. Thus, he had high expectations of the UK, and this is expressed in his recollection of arriving in London:

I go to a land where every man is treated equally, the land of the Queen. Okay … I don’t care, but at least there a minister will resign if they catch something on him, so I learned you can twist the law but don’t break it because you will pay and I like that. One rule about everyone …

Pipo expresses the view that in the UK, in contrast to in Algeria, a politician would be held to account for corruption. When Pipo took his British citizenship test, he had to swear allegiance to the monarch, which afforded him a sense of purpose, arguing ‘you live for such a thing because if there is not such a rule, we would be like zombies, you know?’.

In the accounts provided by Angelina, Joshua and Pipo, we see stranger views of the UK from those who, in many cases, have been in the country for some time. They are not newcomers and have long been exposed to colonial imaginaries of Britain. Their views of Britain told them that this was a place where they could belong, where they could trust the system and feel they were part of something ‘more civilised’ and ‘more developed’. Their views and sense of belonging contain a degree of colonial‐tinged idealism and, as we will show in the next section, co‐exist with experiences of destitution and thus articulate a simultaneous sense of belonging and non‐belonging.

### 5.2 Experiences of the Hostile Environment

In her reflections on border control, Bhattacharyya (2018, 127) notes that it does not serve to block all movement; rather, it serves as a filtering mechanism that enables differential access to the labour market. This exposition of racial capitalism (Tazzioli and de Genova 2023) explains how the contradictory tendencies of nationalist ‘hard border’ policies such as Donald Trump's professed desire to create a Mexico‐United States border wall or Theresa May's avowal to ‘create, here in Britain, a really hostile environment for illegal immigrants’ (cited in Griffiths and Yeo 2021) coexist with the need for the countries such as the US and UK to maintain a supply of cheap labour. This tiering process racializes individuals as migrants and serves to perpetuate the unequal distribution of capital across the social system (Meghji 2021).

The accounts we present here belong to individuals that were placed in spaces of non‐belonging by the immigration system (Korteweg and Yurdakul 2024). The experiences of these spaces are highlighted in the affective responses of migrants to the bordering they have to navigate. In addition to the external borders, and the visible threat of deportability posed by the ‘border spectacle’ (De Genova 2002), migrants experiencing homelessness are forced to negotiate the internal borders of the hostile immigration environment. For example, in the UK (and most of Europe), secondary immigration control leads to ‘everyday bordering’, which is where teachers, landlords, employers and support workers have to perform the role of de facto border guards, checking the paperwork of their clients (migrants or not) and rendering them liable if they offer services to ‘illegal’ migrants (Yuval‐Davis et al. 2018). As Back et al. (2012) remind us, the socio‐legal and political mechanisms of bordering are enforced through a hierarchy of belonging and deservingness, sustained by fear (p. 151).

In the previous section, we mentioned Joshua's Anglophilic stance. Here we show how it co‐exists with his experiences of the UK's hostile environment relegating him to places of non‐belonging. He acknowledges that ‘the Home Office destroyed my life. And I don't deserve this, I caused no harm to nobody in life’. Joshua's experiences are a testament to the vulnerable position migrants are placed in by the UK immigration system. Before he became homeless, he had been living and working in the UK for several decades. During his time as an undergraduate, he was informed that the student visa application that he had submitted to the Home Office had been received a day late and he was thus ineligible to remain in the UK. He contested the decision and was ultimately successful in this case. However, dealing with the uncertainty and dense bureaucracy of the immigration system disrupted his studies. Despite this, he managed to secure part‐time employment and had a successful period of work that lasted until he was made redundant during a period of restructuring at his organisation. At this stage, the NRPF condition on his immigration status kicked in and when he lost his accommodation, he joined the ranks of the ‘hidden homeless’ and, for a period of time, stayed with friends and sofa‐surfed. After he felt that he had outstayed his welcome, he started to practice what we term ‘cultivated invisibility’, which refers to a habitual condition whereby an individual learns to blend into the crowd (or avoids the crowd) in order to find places to sleep (such as on buses during the morning rush‐hour), to eat (e.g., finding the cheapest sources of fast‐food) and wash (registering in gyms or using the facilities at train stations) (Stewart and Sanders 2023). In the meantime, as he moved unnoticed among the early morning commuters, he drifted further towards illegality and ill health: without the correct immigration paperwork, he was vulnerable to deportation. In line with Wacquant’s (2008, 241) notion of advanced marginality, Joshua was forced to be ‘on the move’ and was deprived of a specific locale which could be characterised as ‘home’. Without access to housing or welfare, his health deteriorated rapidly and he was not able to get the requisite care. With his case expressive of advanced marginality, Joshua had no hinterland, no community to fall back upon for support.

We also mentioned Pipo before, and noted his trust of the British legal system. Having been granted refugee status, he was able to build a successful career in hospitality and was happily married. However, the Global Financial Crisis hit his industry hard and he was unable to sustain stable work. He grew increasingly depressed, struggling from insomnia and recollections of the violence he had witnessed in Algeria. With the pressures bearing down upon him having lost his job, he split up with his wife and lost his home, in a short period of time. In later years, having struggled to find secure accommodation and work, he was sleeping in a local park. The legal system he had trusted was not able to protect him when he needed it most.

The Home Office rejected Angelina's application for asylum on the grounds that she did not face a serious enough threat and was considered to be in good health and thus not in need of urgent support. This had drastic consequences as she fell into destitution, sleeping rough, without recourse to benefits, and without any kind of state support. She relied on small donations from some charities. When she tried to make an appeal in relation to her application for asylum, Angelina found out that her files had been lost on the system. It took more than a year for her case to be considered. Angelina contrasts her experiences of being a tourist and an asylum seeker and reflects that her initial failure to obtain refugee status was due to her lack of knowledge of the system and of English legal language:

London is a big city for shopping just only, but when I came here as asylum seeker to build a life from nothing and to go from the public services and to deal with this. I don’t have that knowledge of English words. When you go in [for an interview with the Home Office] this is something different than to come for shopping to choose a dress this colour … it is completely different, or to eat this food, to taste this ice cream. It is something different. But here you came and you face with the government sector cases and appeals and laws. It is horrible.

Arian, an Albanian man in his mid‐forties, arrived in the UK more than 2 decades ago, fleeing a family feud that threatened his life. His engagements with the UK's Home Office also provide a sobering account of the detrimental effects of the UK's immigration system when contrasted with the years in which he seemingly ‘belonged’ in the UK. When he arrived in the UK, Arian sought help at the Immigration and Nationality Directorate (as it was called at the time) who provided him with accommodation in various locations. Arian secured immigration status after some rejections and found work. Soon, he was married and living a fulfiling life. Some 20 years later, he submitted his application a few days late in error and his visa was revoked with devastating consequences. First, Arian lost his right to work, and so his employer was forced to let him go or face a substantial fine if he continued to employ him. He also lost the right to access welfare support including housing benefit and access to social housing. Arian and his wife were evicted from their council property and could not afford to rent in the private sector. These financial strains contributed to the breakdown of his marriage. Arian lost touch with his friendship network and experienced a swift decline into homelessness. The situation demonstrated the precarious position that he occupied on the margins of society as a consequence of his immigration status despite being a long‐term resident of the UK with a stable income and housing situation.

At the time of our interview, Arian was still awaiting a Home Office decision on his claim for indefinite leave to remain having lived and worked (and paid taxes) in the UK for more than 2 decades. He notes with desperation that he is not able to find any updates on his case. This led to an absurd situation whereby he travelled to Croydon to hand himself in for deportation but was told that his case was pending and therefore he could not be deported. We see in this example how a liminal state of non‐belonging is fostered by state practices and how this is embodied in Arian's palpable sense of desperation (Korteweg and Yurdakul 2024):

… when they process your application, they don’t give you updates on it, they write you once to say they’re dealing with it, and your case is active, but after that wait for decision, because if you try to call them you can’t, it’s no good, they say, but won’t say why … you’re waiting, waiting, waiting … all three years, and then I say, no, I had enough, I’m going home. And last year, it was after the COVID laid off a bit, I went to Croydon and said, I’m going home, I [want to be] detained. [They replied] ‘We can’t detain you because you’re not illegal, and you’re not legal’.

We see in the case of Arian the Home Office's active production of a condition of non‐belonging that is experienced by those waiting for the permission to build or rebuild their lives in the UK. It also highlights the fragility of the belonging that accompanied his life in the UK during the time of his marriage and experiences of stable work.

The accounts provided in this section, by Joshua, Pipo, Angelina and Arian offer insights into the immigration system from the perspective of those who are at its mercy. Their stranger views are expressive of the frustration that occurs when individual qualities are disregarded in favour of legal categories that fix them in spaces of non‐belonging.

## 6 Conclusion

This article draws attention to the double meaning of our notion of ‘stranger views’, which are expressive on the one hand, of the experiences of those who occupy a marginal position in society characterised by experiences of belonging and non‐belonging; and on the other, of our own position as researchers, probing spaces of non‐belonging and hearing stories that are then rearticulated for an academic audience. Neither of the authors of this article has ever experienced homelessness or precarious, insecure housing conditions. Thus, in depicting lives very different from their own, the authors potentially contribute to the perpetuation of the participants' marginal status. Who gets to do research about the margins? Who gets to tell the stories of marginalised communities and who benefits from this storytelling? Unfortunately, difficult questions often remain unanswered. Nevertheless, we have used our own research practice to ask these questions and open a conversation about reflexivity in the context of doing research on marginality. We have done so by bringing our own positionality to the fore, and subjecting our own research practice to the scrutiny of the New Area Studies framework. The double‐edged nature of the knowledge we produce is further exemplified by the potential consequences of trying to effect positive change through academic research. For example, a pathway to impact has been to find ways of turning a research finding, that is, a lack of immigration advice leading to increased risk of destitution, into an outcome, that is, the increased provision of immigration advice for migrants experiencing homelessness leading to resolved statuses and access to resources. However, as we have seen above, legal categories associated with immigration status are sources of exclusion as much as inclusion and attempts to resolve statuses require operating within the existing rules which accord with the interests of the dominant.

What is the value of this research? We argue that the life stories produced in the course of this research have the potential to inform social change as they enable insights into systems of domination from the perspectives of those most impacted by these systems. In connecting individual stories to wider social structures, it is possible to trace social causes of homelessness, destitution and non‐belonging. In this article, we have tried to show both that we are aware of the pitfalls of working with marginalised communities, while also recognising the emancipatory power of stories from the margins. New Area Studies approaches invite us to critically reflect on the organising concepts of our research and to include our own positionality in that reflection. This is what doing ‘power‐critical research’ entails. The purpose of this reflection is to be explicit about the challenges of producing knowledge when working with marginalised communities and the implications of operating within existing hierarchies of knowledge (Barrios Aquino 2024).

Our research points to the potential inherent in the analysis of stories of people and places in envisaging alternative futures. What might such futures hold? Sociological analysis of life stories connects objective conditions of marginality to subjective experience and this is the starting point in any programme to alleviate suffering. The diagnosis precedes the cure. Life story narratives express a ‘relationship between tellers and listeners and their cultural, political and historical contexts’ (Shuman 2010, 25). Whilst stories can be ‘curated’ by institutions to align with dominant ideologies of individualism, they also have the potential to ‘express the fullness and complexity of experience’ and undermine power relations (Fernandes 2017, 4). Life stories enable us to trace the social causes of suffering, as migrants are caught between neoliberal and nationalist policies, and our sociological imagination can proceed to envisage a future where, in a rich country such as the UK, there is no destitution or homelessness. Marginality can indeed be a site of radical possibility (hooks 1991). Whilst anti‐migrant rhetoric and hostile environment policies from political leaders create divisions, life story‐based research which draws attention to our common humanity enables us to imagine ‘the possibility of living together differently, with less misery or no misery’ (Bauman 2000, 215).

This article offers a version of Life in the UK that is directly in conflict with hegemonic views presented in the knowledge of life and language required by the British immigration regulations. We have acknowledged that we are strangers to the lives of those we have researched just as the research participants have offered their own stranger views, articulating a sense of belonging to the UK based on their longstanding residency in the country, their active participation as workers, partners, friends, and members of the community, and their positive views of the UK, alongside a sense of non‐belonging deriving from a lack of access to resources and opportunities, experiences of homelessness and destitution, and the consequences of liminal immigration statuses. In this sense, we, the authors, as much as the migrants whose stories we presented here, are like Simmel's strangers.

## Ethics Statement

This research was approved by the University of Portsmouth Ethics Committee (Reference Number: FHSS 2020‐053). The data have been anonymised and the names used in this article are pseudonyms chosen by the respondents.

## Conflicts of Interest

The authors declare no conflicts of interest.

## Data Availability

Due to ethical requirements, data sharing is not possible for this article.

## Endnotes

^1^
Grant reference: ES/V011081/1.
